# Clinical research and burnout syndrome in Italy – only a physicians’ affair?

**DOI:** 10.1186/s13063-021-05158-z

**Published:** 2021-03-12

**Authors:** Celeste Cagnazzo, Roberto Filippi, Giulia Zucchetti, Rosita Cenna, Cristiana Taverniti, Agata Sue Ellen Guarrera, Stefano Stabile, Irene Federici, Manuela Monti, Sara Pirondi, Sara Testoni, Franca Fagioli

**Affiliations:** 1 Unità di Ricerca e Sviluppo Clinico SC Oncoematologia Pediatrica, AOU Città della Salute e della Scienza, Presidio Ospedaliero Infantile Regina Margherita, Piazza Polonia 94, 10126 Turin, Italy; 2 Dipartimento di Scienze della Sanità Pubblica e Pediatriche, Università degli Studi di Torino, Turin, Italy; 3 Gruppo Italiano Data Manager – CRC, Meldola, FC Italy; 4 Oncologia Medica 1, AOU Città della Salute e della Scienza, Presidio Molinette, Turin, Italy; 5 Terapie Cellulari e Medicina Trasfusionale, AOU Careggi, Florence, Italy; 6 SC Oncologia Falck, Niguarda Cancer Center, Grande Ospedale Metropolitano Niguarda, Milan, Italy; 7 Clinica di Ematologia, AOU Ospedali Riuniti Umberto I, G.M. Lancisi, G. Salesi, Ancona, Italy; 8 Biostatistics and Clinical Trials Unit, IRCCS Istituto Scientifico Romagnolo per lo Studio dei Tumori (IRST) Dino Amadori, Meldola, FC Italy; 9 UOSD Oncologia, AUSL Modena Area Sud Ospedale di Sassuolo (MO), Modena, Italy

**Keywords:** Burnout, Clinical research, Depersonalization, Emotional exhaustion, Professional achievement, Italy

## Abstract

**Background:**

The burnout phenomenon has been extensively investigated among health care professionals, particularly focusing on physicians and nurses. However, literature concerning burnout in clinical research is poor and often neglects the other professional categories involved.

**Methods:**

In March 2019, all members of Italian Group of Clinical Research Coordinator were invited to participate to a web survey, consisting of three sections: general information and workload; Maslach Burnout Inventory (MBI) test; subjective perception of oneself’s work stress and possible causes.

**Results:**

The majority of respondents felt a form of distress. The main source was contract type (31.2%), followed by workload (20.5%) and lack of skills recognition (17.8%).

Results from MBI test confirmed the interviewees’ subjective perception: an intermediate level of emotional exhaustion (19.1 points) and a very high sense of reduced professional achievement (26.8 points) were observed. Both depersonalization and sense of reduced professional achievement showed weak to moderate correlations with emotional exhaustion. Emotional exhaustion was associated with contract type with high significance.

**Conclusion:**

It is necessary to act on those qualitative factors that are greatly increasing the level of perceived stress, jeopardizing the quality of clinical research coordinators work and significantly amplifying the phenomenon of migration towards the private sector.

**Supplementary Information:**

The online version contains supplementary material available at 10.1186/s13063-021-05158-z.

## Background

The term “burnout” was coined by psychologist Herbert Freudenberger in 1974 in the article “Staff Burnout,” in which he discussed job dissatisfaction caused by professional stress [1].

There is no standard definition of burnout, but, currently, the most accepted and widespread is the one given by Maslach and Jackson, describing burnout as a tridimensional psychological syndrome, in which professionals who provide human services display emotional exhaustion, depersonalization in client attention and feelings of low personal accomplishment. Put more simply, a job-related state of physical and mental exhaustion, where coping methods are not sufficient [2].

The first burnout dimension is the emotional exhaustion, which refers to stress feelings as depression, hopelessness, anger, loneliness, irritability, impatience, tension, decreased empathy, a sense of lack of energy, and worry. The second dimension, defined as depersonalization, is the tendency to put distance from the recipients of the service, accompanied by a sense of alienation, as well as callousness and indifference towards others, which in turn gives way to cynicism; consequently, working with other people is often considered unpleasant and undesirable. Finally, the feeling of low professional achievement or low job satisfaction (third dimension) can be described as the awareness that very little has been attained and what is accomplished is worthless [2, 3].

Burnout syndrome is responsible for undesirable consequences, both in personal and professional spheres, affecting not only workers themselves, but also their families, the work environment, and the organizations [4]. A variety of psychosomatic and organic health issues are influenced by burnout [5].

Furthermore, burnout has been associated with substance abuse, depression and suicide, as well as medical errors, professional misconduct, departure from the oncology profession, and early retirement [6–11].

Burnout is considered one of the most important occupational health problems in various professions involving working with other people [12] and it is listed in the International Statistical Classification of Diseases and Related Health Problems, 10th revision (ICD-10), under the category “Problems related to life-management difficulty” [13].

The phenomenon of burnout syndrome among health care professionals has been extensively investigated, predominantly focusing on physicians [7, 8, 14–20] and nurses [21–30]. However, literature concerning burnout in clinical research, with particular regard to the professional figures dedicated to its coordination and management, including Clinical Research Coordinators (CRCs), is poor and often referred to a work environment far away from the Italian one.

Mueller and Mamo in 2002 reported three major critical areas faced by CRCs: work autonomy/control, relationship with patients and physicians, and clinical or technical skills and knowledge [31]. Höglund et al. [32] showed CRCs’ ethical dilemmas, such as conflict between their obligations to clinical trials and those to participants. Other studies highlighted issues with multitasking, feeling of isolation, job control, workload, working with patients, collaboration with other healthcare professionals, and uncertainty towards career development [33–36]. Furthermore, Gwede et al. [37] compared burnout levels in CRCs to those reported by other healthcare professionals and Matsumoto et al. [38] tried to validate a psychometric testing for a CRCs Stressor Scale.

However, at the time and place in which these studies were conducted, the figures of the research nurse and the CRC were overlapping to a certain extent, while nowadays in Europe, especially in the field of oncology research, these are two distinct professional figures, with very different duties and stressors.

In most of the Italian trial centers, CRC’s daily work involves close interaction with principal investigators, sub-investigators, nursing staff, sponsors, contract research organizations, ethical committees, and national competent authorities, to ensure that clinical trials are carried out correctly in terms of patient compliance, study procedures, collection of the data, and administrative and other study-related issues. CRC activities are wide ranging and include administrative and regulatory compliance, protocol management, data collection, case report form (CRF) completion and query resolution, serious adverse event reporting, and activity monitoring. However, CRC workload varies substantially among Italian and European institutions, mainly because of the lack of common guidelines on the role and duties of this professional figure. Few articles on the activities of a CRC and on the number of studies in which CRCs are involved are available on MEDLINE, and all vary substantially in the information they provide [39].

Due to this substantial lack of knowledge, the Italian Group of Clinical Research Coordinators (GIDM) conducted the present study, based on a survey among its associates aimed at investigating the prevalence of self-reported stress and burnout symptoms in CRCs working in clinical research, as well as evaluating the applicability of a standardized tool such as the Maslach Burnout Inventory (MBI) in this population.

## Methods

In March 2019, all CRC members of GIDM, at that time accounting for 196 members, were invited to participate in the survey, via an email containing the link to complete the questionnaire; all invitations were sent simultaneously, through a mailing list. Participation was voluntary; no reward was offered, nor a fee was requested, for completing the survey. The participation link was active for 15 days following the email receipt.

The survey required 15 min to complete and consisted of three sections:

- Section 1 (general information and workload): 7 questions regarding the information of respondent (e.g., type of workplace, years of experience, contract type), and the number and status (ongoing or closed enrollment) of attended studies.
- Section 2: the MBI form.
- Section 3 (personal experience): 7 questions about the subjective perception of oneself’s work stress and possible causes.

The English version of the survey is reported in supplementary Appendix S1.

Before producing the final version of the survey, an initial draft (version 0) was delivered as a preliminary test to 36 CRCs from 4 hospitals, 4 IRCCs, and 4 universities. The comments and corrections collected were implemented giving way to a new version (version 1) delivered to 9 more CRCs with homogeneous distribution among the different types of institutes. This new test represented the final version of the questionnaire used in this project.

The survey did not include fields for identification of participants, including information on age and sex, or their specific institution. Collection of surveys, calculation of results of Section 2, and data entry on a password-protected electronic database were performed by a third operator who had no access to information regarding GIDM members’ emails addresses or identities.

Given the descriptive nature of the survey, we did not formally estimate a required sample size, the study being aimed at collecting the largest number of experiences possible. To this extent, the lack of a formally defined professional figure and therefore of a register of known professionals makes it impossible to fully know the total number of CRCs in Italy and their regional and inter-regional distribution.

However, considering the pilot nature of the study and the potential issue of representativeness with an excessively small sample, performing data analysis was conditional to reaching a preplanned minimum threshold of respondents set at 30% of the total population of GIDM members. Data were analyzed in December 2019. Descriptive statistics and Pearson coefficient for correlational analysis were employed. Kolmogorov-Smirnov test was employed as normality test; Student’s *t* test was employed to assess the statistical significance of the observed differences. SPSS Statistics® v.20 (IBM Analytics) was employed as main statistical program.

## Results

The survey was completed by 57.1% (*n* = 112) subjects, thus allowing the subsequent data analysis. A large majority (85%) of the twenty Italian regions were represented, with a prevalence of participants from Northern regions: Lombardy (*n* = 37, 33.0%), Emilia Romagna (*n* = 16, 14.3%), and Piedmont (*n* = 11, 9.8%).

Most of the respondents (58.9%) worked in public hospitals/local health departments/universities, followed by private (22.3%) and public research institutes (11.6%); a small minority was employed in private hospitals (7.2%). Almost all respondents worked in hematology and oncology fields (*n* = 109).

The level of experience of the interviewees was heterogeneous: 46.4% had been working for less than 5 years, 34.8% from a period between 5 and 10 years, and 18.8% from more than 10 years.

At the time of the interview, only a few respondents could rely on a stable contract, permanent- (21.4%) or fixed-term (12.5%), while most (66.1%) worked through diverse temporary contracts (e.g., freelance, or project-based contracts).

The average number of studies followed by the interviewees was 12.4 actively enrolling trials, and 10.6 trials closed for recruitment.

The majority of respondents (85.7%) felt a form of distress; moreover, 67.7% of them (*n* = 65) claimed that such stress adversely affected work performance and 73.9% (*n* = 71) was thinking of changing job. The main cause of perceived stress was contract type (31.2%), followed by workload (20.5%) and lack of skills recognition (17.8%).

Results from MBI confirmed the interviewees’ subjective perception. While the risk of depersonalization was low (mean 4.3 points), an intermediate level of emotional exhaustion (19.1) and a very high sense of reduced professional achievement (26.8) were observed. Emotional exhaustion numerically correlated with job duration (mean value 19.4 for < 5 years of experience, 21.5 for 5–10 years, 25.9 for > 10 years), which translated into a statistically significant difference between < 5 years and >  10 years categories (*p* = 0.013). The trend was confirmed by linear regression analysis (*p* = 0.022). Furthermore, emotional exhaustion significantly correlated with contract type, with atypical workers self-attributing lower scores than workers subject to regular contracts (19.6 vs 24.8, *p* = 0.025).

Depersonalization and sense of reduced professional achievement did not differ according to job duration. Instead, depersonalization scores were higher among interviewees working in the private sector (5.7 vs 3.6 among those working in public sector, *p* = 0.048), and higher scores in the sense of reduced professional achievement were self-attributed by interviewees working in hospitals (28.3 vs 23.4 by those working in research centers, *p* = 0.033) (Table 1). Both depersonalization and sense of reduced professional achievement, which lacked mutual correlation, showed weak to moderate correlations with emotional exhaustion (Table 2). Patient-level MBI scores are listed in Appendix S2.

**Table 1:** Subgroup analysis of mean Maslach Burnout Inventory (MBI) values and their standard deviation (SD)

	*n*	Emotional exhaustion		Depersonalization		Sense of reduced professional achievement	
		Mean MBI (SD)	*p*	Mean MBI (SD)	*p*	Mean MBI (SD)	*p*
*Job duration (years)*							
< 5	52	19.4 (11.6)	0.106	3.9 (4.1)	0.234	26.7 (11.9)	0.901
5 to 10	39	21.5 (11.6)		5.2 (6.8)		26.1 (11.1)	
> 10	21	25.9 (11.3)		3.0 (3.3)		27.5 (12.1)	
*Workplace*							
Public	79	20.9 (11.3)	0.509	3.6 (4.0)	0.048	27.8 (11.1)	0.103
Private	33	22.5 (12.5)		5.7 (7.0)		23.9 (12.3)	
Hospital	74	21.2 (11.4)	0.831	4.6 (5.5)	0.280	28.3 (11.0)	0.033
Research center	38	21.7 (12.4)		3.5 (4.1)		23.4 (12.0)	
*Contract*							
Permanent	24	24.2 (11.3)	0.181	5.6 (7.5)	0.131	24.7 (12.3)	0.351
Temporary	88	20.6 (11.7)		3.8 (4.3)		27.2 (11.4)	
Typical	38	24.8 (11.5)	0.025	4.8 (6.4)	0.379	25.9 (11.7)	0.635
Atypical	74	19.6 (11.4)		3.9 (4.3)		27.0 (11.5)

**Table 2:** Correlations among features of burn-out syndrome

Correlation	Pearson correlation coefficient	***p*** value
Emotional exhaustion and depersonalization	0.340	< 0.001
Emotional exhaustion and sense of reduced professional achievement	0.231	0.014
Depersonalization and sense of reduced professional achievement	0.103	0.281

A significant minority of interviewees (14.2%) reported that at least one MBI test question was not applicable to them; all answers considered not applicable for the type of respondents concerned the relationship between the professional and the patient.

## Discussion

Historically, stress levels among healthcare professionals have been attributed to the relationship with the patient. For this reason, literature shows burnout data mainly for physicians and nurses.

A preliminary investigation conducted among GIDM members had already suggested that burnout could be a scourge for other categories as well [40]. Results of that internal pilot study, conducted without a validated test, were confirmed by the present investigation: perceived stress was largely prevalent in the studied sample and frequently led respondents to consider a career change.

Such alarmingly negative perceptions resonated in the MBI scores: if the risk of depersonalization was low, the levels of emotional exhaustion and sense of reduced professional achievement were, more worryingly, intermediate and high, respectively. In the examined population, these features of burnout showed a degree of inter-relation, most prominent for emotional exhaustion, which correlated with a number of job-related variables. Particularly, our work suggested an increase in emotional exhaustion with job duration, but the threshold for statistical significance was reached only with further, ad hoc testing. This could be due to the small size of the subgroups: particularly, workers with > 10 years of working experience were only 21. This correlation is interesting, suggesting a time-dependent wearing nature of the job-related stressors, and may deserve appropriate validation in a larger, prospective, longitudinal study.

The present study provides preliminary evidence in a largely unexplored field, with paucity of pertaining existing literature. However, a number of limitations should be acknowledged.

A significant proportion of GIDM members is represented by CRCs working at large academic medical institutions, whereas CRCs who work at smaller research sites are under-represented [41]. The observed low rate of response, unfortunately a common issue in survey-based research, resulted similar to that registered by Gwede et al. in a similar US population [37]. To protect respondents’ privacy, no demographic or workplace information were collected.

The MBI was not specifically designed for a professional figure such as the CRC, with its own peculiarities that differentiate it from similar positions, and that has been displaying a dynamic and evolving nature since its relatively recent appearance. For instance, the test does not take into consideration of the main source of dissatisfaction, which is contract type: in Italy, CRCs are often hired with precarious contracts [41, 42], lacking a definition of tasks and duties. Tellingly, nearly half of respondents were professionals with less than 5 years of working experience. Given the focus on the national context, presenting with the mentioned peculiarities, it is unlikely that the conclusions of this study can be generalized for CRCs working in other countries.

The lack of a recognition stems from the absence of a formal definition of this specific professional role, along with the need for a professional register, a very common condition in European countries with very few exceptions. Indeed, research on this new field needs shared and validated tools for evaluation. As further proof of the fact that these unmeasurable factors appear to be the main driver of CRC work stress in Italy, no clear connection of stress with workload was observed, contrarily to Gwede et al.’s [37] observations. Of course, that does not mean that excessive workload is not a widespread phenomenon in this field, but probably that the number of expected studies resulted in a generic, unreliable approximation of workload, failing to capture the substantial differences in work intensity that exists between active studies and studies in follow-up, observational and active treatment studies, and no-profit and profit trials. Furthermore, the arrival of new study designs (e.g., basket, umbrella, and adaptive studies) makes it even more difficult to identify a unique method for an accurate workload estimate the workload. These observations highlight the need for more refined, standardized measurements of workload in this field, factoring in more variables than the mere number of weekly worked hours and attended studies.

## Conclusion

In the first study ever conducted on burnout among Italian CRCs, despite the near totality of high levels of stress perceived, psychological testing showed a medium-low degree of burnout intended as depersonalization and emotional exhaustion. Burnout was substantially uncorrelated to quantitative estimates of workload, rather depending on other, qualitative, factors, such as lack of skills recognition and contractual instability.

It is therefore not surprising that the only sphere of burnout to record very high scores is that relating to job dissatisfaction.

Our data suggest that current workload measurement methods, mainly based on the number of working hours and attended studies, are no longer appropriate for a multi-faceted, evolving job and need to be overcome for the purpose of future research. Our observations lay the foundations for more refined and appropriate evaluation in a largely unexplored field that should rely on more complex and validated tools and involve international collaboration.

One fact remains clear: it is necessary to act on those qualitative factors that are greatly increasing the level of perceived stress, significantly amplifying the phenomenon of migration to private companies that has already reached worrying levels.

## Clinical implication

Ashill and Rod [43] identified a close relationship between job demand stressors (work overload, role conflict, role ambiguity, and interpersonal conflict), symptoms of burnout (emotional exhaustion and depersonalization), affective job outcomes (job satisfaction and organizational commitment), and behavioral job outcomes (service recovery performance and turnover intentions). The authors also stressed the importance of extending our understanding of these phenomena in the largely unexplored yet important context of non-clinical health professionals. The major implication for hospital managers is to ensure that non-clinical healthcare workers have adequate information pertaining to their job-related duties and responsibilities, since role ambiguity resulted from the only characteristic of the non-clinical work environment that influenced subsequent appraisal (depersonalization), emotional response (organizational commitment), and behavior (service recovery performance) [43].

Ofei-Dodoo et al. [44] investigated job satisfaction and burnout among non-clinical workers in a US medical education center: 1% of the 95 respondents reported high burnout and 35% reported medium burnout. Despite these concerning statistics, the burnout rates of the sample were better than that of the general population of US workers (excluding physicians), where 53% reported symptoms of burnout. The authors concluded that non-clinical workers who are satisfied with their job reported a low degree of burnout and, in particular, job satisfaction negatively correlated with all three dimensions of burnout. In addition, they suggested that negative feelings about one’s job, poor relationships with colleagues, lack of support, and lack of teamwork with coworkers were responsible for burnout among non-clinical workers [44].

The findings of these exploratory studies highlight the importance of job satisfaction factors among non-clinical workers such as nature of work, support from coworkers, supervision, and contingent rewards. Both job satisfaction and burnout constructs are well studied among clinicians, while burnout and its consequences are understudied among the population of our research, CRCs [45].

Burnout seems to occur mainly in professions involving an interaction with people. Symptoms of burnout negatively affect quality of life, which in turn may prompt the affected person to look for help and intervention. The list of symptoms is long and most of them are not very specific. These include warning symptoms in the early phase (increased commitment to goals and exhaustion), followed by a phase of reduced commitment (towards patients and clients, towards others in general, towards work, towards increased demands), emotional reactions and blaming (depression, aggression), and finally leading to reduction in cognitive performance, motivation, creativity, and judgment; flattening of emotional, social, and intellectual life; and psychosomatic reactions and despair. Consequences of burnout are decreased job satisfaction, absenteeism, turnover in personnel, and cynicism. These effects at work frequently have repercussions on personal life such as feeling unhappy, anxiety, depression, isolation, substance abuse, frictional and broken relationships, and divorce. Even if the effect of burnout among the CRCs was not investigated, we can assume that negative implications are not different: worse job performance, depression, abandonment of the job (with loss for the company of expertise and a continuous and resources-consuming turnover), and sick leave.

In a recent review, Williams [46] investigated the differences between burnout and moral injury and discussed the implications for clinicians using electronic health record (EHR), highlighting the need for a better delineation of the problem. Current efforts to improve the EHRs have focused on improving the user experience to reduce burden that has been identified as a contributing factor to burnout [46]. CRCs are also professionals who manage the EHR. Heavy stress increases the risk of error in this context. Predicting the effects of stress on this type of healthcare professional could improve the performance of clinical research in Italy.

## Study limitations

A number of limitations should be acknowledged. Among them are the underrepresentation in our sample of workers from small research sites, low rate of response to the survey, limited sample size, the use of an assessment tool not validated in the CRCs population, and the national context of the study.

## Supplementary Information

**Additional file 1.**

**Additional file 2: Appendix S2.** Complete list of MBI scores collected.

## Data Availability

All data reported in this paper.
