# Supplementary material for: Clinical research and burnout syndrome in Italy – only a physicians’ affair?
Source: Trials. 2021 Mar 12;22:205. doi: 10.1186/s13063-021-05158-z (PMC7953807; doi:10.1186/s13063-021-05158-z)
Supplement: Supplementary file 1 — Additional file 1. [file 13063_2021_5158_MOESM1_ESM.docx]

**APPENDIX S1**

1. In which region are you currently working? *(open question)*

2. What kind of institution are you working in?

1. Public hospital/ local healthcare centre/ university
2. Private hospital
3. Public IRCCS
4. Private IRCCS

3. How many years have you been working?

1. <5
2. 5-10
3. >10

4. What kind of employment contract are you in?

1. fixed term contract
2. permanent contract
3. atypical contract (VAT license, temporary contract, scholarship, research grant)

5. How many studies with open recruitment are you responsible for?

6. How many studies with close recruitment are you responsible for?

7. Do you believe your work is stressing?

1. Yes
2. No

8. Which of the following items is the greatest source of stress for you?

1. Work burden
2. Failure to acknowledge personal and working skills
3. Kind of employment contract
4. Organization of work burdens
5. Working hours per week
6. Human relations with colleagues
7. Human relations with heads of the unit

9. Do you believe your stress level may negatively affect your working output?

1. Yes
2. No

10. Do you believe your stress level may prompt you to look for another job?

1. Yes
2. No

11. BURNOUT TEST

For each of the following questions, select one answer from 0 to 6. Remember that:

0 never

1 few times a year

2 once a month or less

3 few times per month

4 once a week

5 few times a week

6 everyday

1. I feel emotionally exhausted because of my work

2. I feel exhausted at the end of a working day

3. I feel tired when I get up in the morning and I have to face another working day

4. I can easily understand how my patients feel

5. I feel to be dealing with some of my patients as if they were objects

6. I feel tough to work with other people all day long

7. I deal with the problems of my patients effectively

8. I feel drained because of my work

9. I believe to positively affect the life of people through my job

10. Since I started working here, I became more insensitive to people

11. I fear that this work may harden me emotionally

12. I feel myself full of energies

13. I am frustrated because of my job

14. I believe to be working too hard

15. I do not really care about what happens to some of my patients

16. Working directly with people makes me too nervous

17. I easily make my patients fell at ease

18. After working with my patients, I feel cheered up

19. I accomplished many valuable things in my work

20. I feel to I cannot take it anymore

21. In my work, I calmly face emotional problems

22. I feel my patients blame me for some of their problems

12. Comments: *(open question)*
