# Supplementary material for: Clinical research and burnout syndrome in Italy – only a physicians’ affair?
Source: Trials. 2021 Mar 12;22:205. doi: 10.1186/s13063-021-05158-z (PMC7953807; doi:10.1186/s13063-021-05158-z)
Supplement: Supplementary file 2 — Additional file 2: Appendix S2. Complete list of MBI scores collected. [file 13063_2021_5158_MOESM2_ESM.docx]

**Appendix S2: Complete list of MBI scores collected**

| **Interviewed** | **Emotional exhaustion** | | **Depersonalization** | | **Sense of reduced professional achievement** | | \| **Years of expertise** \|  \| \| --- \| --- \| | **Workplace type** | **Contract type** |
| --- | --- | --- | --- | --- | --- | --- | --- | --- | --- | --- | --- |
|  | **Points** | **Range** | **Points** | **Range** | **Points** | **Range** |  |  |  |
| 1 | 21 | MEDIUM | 3 | LOW | 38 | MEDIUM | < 5 | Public Hospital | Unstable |
| 2 | 27 | MEDIUM | 1 | LOW | 31 | A | 5-10 | Public Hospital | Unstable |
| 3 | 4 | LOW | 0 | LOW | 11 | A | < 5 | Private Research Centre | Unstable |
| 4 | 30 | A | 0 | LOW | 38 | MEDIUM | > 10 | Private Research Centre | Permanent |
| 5 | 12 | LOW | 2 | LOW | 20 | MEDIUM | < 5 | Public Hospital | Unstable |
| 6 | 16 | LOW | 8 | MEDIUM | 27 | A | 5-10 | Private Research Centre | Unstable |
| 7 | 17 | LOW | 7 | MEDIUM | 42 | LOW | < 5 | Public Hospital | Unstable |
| 8 | 39 | A | 22 | A | 21 | A | 5-10 | Private Hospital | Permanent |
| 9 | 27 | MEDIUM | 11 | MEDIUM | 34 | MEDIUM | 5-10 | Private Research Centre | Permanent |
| 10 | 19 | MEDIUM | 12 | A | 19 | A | 5-10 | Public Hospital | Permanent |
| 11 | 36 | A | 7 | MEDIUM | 15 | A | < 5 | Public Hospital | Unstable |
| 12 | 10 | LOW | 5 | LOW | 39 | MEDIUM | < 5 | Public Hospital | Unstable |
| 13 | 13 | LOW | 6 | MEDIUM | 25 | A | 5-10 | Private Research Centre | Permanent |
| 14 | 7 | LOW | 0 | LOW | 45 | LOW | < 5 | Public Hospital | Unstable |
| 15 | 18 | MEDIUM | 2 | LOW | 27 | A | < 5 | Public Hospital | Unstable |
| 16 | 32 | A | 5 | LOW | 33 | A | 5-10 | Public Hospital | Fixed-term |
| 17 | 35 | A | 0 | LOW | 37 | MEDIUM | < 5 | Public Hospital | Unstable |
| 18 | 25 | MEDIUM | 2 | LOW | 30 | A | > 10 | Private Research Centre | Permanent |
| 19 | 25 | MEDIUM | 4 | LOW | 21 | A | 5-10 | Private Research Centre | Fixed-term |
| 20 | 14 | LOW | 0 | LOW | 41 | LOW | 5-10 | Public Hospital | Unstable |
| 21 | 37 | A | 5 | LOW | 40 | LOW | > 10 | Private Research Centre | Permanent |
| 22 | 40 | LOW | 6 | MEDIUM | 24 | A | < 5 | Public Hospital | Unstable |
| 23 | 15 | LOW | 15 | A | 43 | LOW | < 5 | Private Research Centre | Unstable |
| 24 | 12 | LOW | 4 | LOW | 45 | LOW | > 10 | Public Hospital | Unstable |
| 25 | 37 | A | 0 | LOW | 40 | LOW | < 5 | Public Hospital | Fixed-term |
| 26 | 30 | A | 0 | LOW | 13 | A | 5-10 | Public Hospital | Permanent |
| 27 | 23 | MEDIUM | 1 | LOW | 30 | A | 5-10 | Public Hospital | Fixed-term |
| 28 | 22 | MEDIUM | 1 | LOW | 20 | A | < 5 | Public Hospital | Unstable |
| 29 | 22 | MEDIUM | 0 | LOW | 29 | A | < 5 | Public Hospital | Fixed-term |
| 30 | 9 | LOW | 1 | LOW | 7 | A | 5-10 | Public Hospital | Permanent |
| 31 | 25 | MEDIUM | 3 | LOW | 40 | LOW | 5-10 | Public Hospital | Unstable |
| 32 | 33 | A | 8 | MEDIUM | 27 | A | > 10 | Public Hospital | Fixed-term |
| 33 | 12 | LOW | 8 | MEDIUM | 21 | A | 5-10 | Public Hospital | Unstable |
| 34 | 23 | MEDIUM | 1 | LOW | 36 | MEDIUM | < 5 | Public Hospital | Unstable |
| 35 | 31 | A | 0 | LOW | 15 | A | < 5 | Private Research Centre | Fixed-term |
| 36 | 32 | A | 8 | MEDIUM | 35 | MEDIUM | < 5 | Public Hospital | Unstable |
| 37 | 42 | A | 30 | A | 35 | MEDIUM | 5-10 | Private Hospital | Permanent |
| 38 | 24 | MEDIUM | 1 | LOW | 18 | A | > 10 | Private Research Centre | Unstable |
| 39 | 32 | MEDIUM | 1 | LOW | 16 | A | > 10 | Private Research Centre | Permanent |
| 40 | 23 | MEDIUM | 2 | LOW | 42 | LOW | < 5 | Public Hospital | Unstable |
| 41 | 49 | A | 0 | LOW | 37 | MEDIUM | > 10 | Public Research Centre | Unstable |
| 42 | 11 | LOW | 2 | LOW | 21 | A | 5-10 | Public Hospital | Unstable |
| 43 | 18 | MEDIUM | 0 | LOW | 11 | A | > 10 | Private Research Centre | Permanent |
| 44 | 11 | LOW | 0 | LOW | 23 | LOW | < 5 | Public Hospital | Unstable |
| 45 | 32 | A | 0 | LOW | 40 | LOW | < 5 | Public Hospital | Fixed-term |
| 46 | 12 | LOW | 8 | LOW | 42 | LOW | 5-10 | Public Hospital | Unstable |
| 47 | 18 | MEDIUM | 8 | MEDIUM | 43 | LOW | < 5 | Public Hospital | Unstable |
| 48 | 33 | A | 11 | MEDIUM | 12 | A | < 5 | Public Hospital | Unstable |
| 49 | 5 | LOW | 0 | LOW | 21 | A | < 5 | Public Research Centre | Unstable |
| 50 | 10 | LOW | 0 | LOW | 14 | A | < 5 | Public Hospital | Unstable |
| 51 | 12 | LOW | 3 | LOW | 38 | MEDIUM | > 10 | Public Hospital | Fixed-term |
| 52 | 40 | A | 9 | MEDIUM | 33 | A | > 10 | Public Hospital | Unstable |
| 53 | 17 | LOW | 2 | LOW | 13 | A | > 10 | Public Hospital | Unstable |
| 54 | 11 | LOW | 0 | LOW | 41 | LOW | < 5 | Public Hospital | Unstable |
| 55 | 11 | LOW | 0 | LOW | 2 | A | < 5 | Private Research Centre | Unstable |
| 56 | 40 | A | 11 | MEDIUM | 29 | A | > 10 | Private Research Centre | Permanent |
| 57 | 35 | A | 0 | LOW | 47 | LOW | > 10 | Public Hospital | Permanent |
| 58 | 18 | MEDIUM | 0 | LOW | 35 | MEDIUM | 5-10 | Public Research Centre | Unstable |
| 59 | 29 | MEDIUM | 0 | LOW | 48 | LOW | 5-10 | Public Research Centre | Unstable |
| 60 | 16 | LOW | 0 | LOW | 28 | A | < 5 | Public Hospital | Unstable |
| 61 | 42 | A | 8 | MEDIUM | 39 | MEDIUM | 5-10 | Public Hospital | Unstable |
| 62 | 41 | A | 0 | LOW | 39 | MEDIUM | 5-10 | Public Hospital | Unstable |
| 63 | 38 | A | 7 | MEDIUM | 33 | A | 5-10 | Public Hospital | Unstable |
| 64 | 3 | LOW | 6 | MEDIUM | 41 | LOW | < 5 | Public Hospital | Unstable |
| 65 | 13 | LOW | 0 | LOW | 17 | A | 5-10 | Public Hospital | Fixed-term |
| 66 | 3 | LOW | 0 | LOW | 34 | MEDIUM | 5-10 | Public Hospital | Unstable |
| 67 | 24 | MEDIUM | 6 | MEDIUM | 43 | LOW | < 5 | Private Hospital | Permanent |
| 68 | 13 | LOW | 2 | LOW | 20 | A | 5-10 | Public Hospital | Unstable |
| 69 | 12 | LOW | 4 | LOW | 19 | A | < 5 | Public Hospital | Unstable |
| 70 | 29 | MEDIUM | 8 | MEDIUM | 30 | A | 5-10 | Public Research Centre | Unstable |
| 71 | 8 | LOW | 6 | MEDIUM | 28 | A | < 5 | Public Hospital | Unstable |
| 72 | 8 | LOW | 0 | LOW | 23 | A | < 5 | Public Research Centre | Unstable |
| 73 | 15 | LOW | 11 | MEDIUM | 26 | A | < 5 | Public Research Centre | Unstable |
| 74 | 35 | A | 16 | A | 26 | A | < 5 | Private Hospital | Unstable |
| 75 | 12 | A | 1 | LOW | 8 | A | 5-10 | Private Hospital | Permanent |
| 76 | 1 | LOW | 1 | LOW | 11 | A | 5-10 | Public Research Centre | Unstable |
| 77 | 9 | LOW | 4 | LOW | 3 | A | < 5 | Private Research Centre | Fixed-term |
| 78 | 24 | MEDIUM | 6 | MEDIUM | 12 | A | < 5 | Public Research Centre | Unstable |
| 79 | 5 | LOW | 6 | MEDIUM | 45 | LOW | < 5 | Private Hospital | Unstable |
| 80 | 14 | LOW | 5 | LOW | 6 | A | 5-10 | Public Hospital | Unstable |
| 81 | 25 | MEDIUM | 13 | A | 22 | A | 5-10 | Public Hospital | Unstable |
| 82 | 28 | MEDIUM | 3 | LOW | 22 | A | > 10 | Public Research Centre | Unstable |
| 83 | 35 | A | 9 | MEDIUM | 35 | MEDIUM | < 5 | Private Hospital | Fixed-term |
| 84 | 50 | A | 10 | MEDIUM | 28 | A | < 5 | Private Research Centre | Fixed-term |
| 85 | 26 | MEDIUM | 1 | LOW | 15 | A | < 5 | Public Hospital | Unstable |
| 86 | 34 | A | 6 | MEDIUM | 44 | LOW | < 5 | Public Research Centre | Unstable |
| 87 | 1 | LOW | 2 | LOW | 12 | A | 5-10 | Private Research Centre | Permanent |
| 88 | 28 | MEDIUM | 2 | LOW | 16 | A | < 5 | Public Hospital | Unstable |
| 89 | 15 | LOW | 2 | LOW | 18 | A | 5-10 | Public Hospital | Unstable |
| 90 | 37 | A | 1 | LOW | 40 | LOW | 5-10 | Public Hospital | Unstable |
| 91 | 3 | LOW | 0 | LOW | 6 | A | > 10 | Public Hospital | Unstable |
| 92 | 34 | A | 3 | LOW | 14 | A | < 5 | Public Research Centre | Unstable |
| 93 | 16 | LOW | 5 | LOW | 19 | A | < 5 | Public Hospital | Unstable |
| 94 | 25 | MEDIUM | 2 | LOW | 24 | A | > 10 | Private Hospital | Permanent |
| 95 | 31 | A | 0 | LOW | 8 | A | 5-10 | Private Research Centre | Permanent |
| 96 | 16 | LOW | 0 | LOW | 19 | A | < 5 | Public Hospital | Unstable |
| 97 | 5 | LOW | 2 | LOW | 25 | A | < 5 | Public Hospital | Unstable |
| 98 | 19 | MEDIUM | 0 | LOW | 33 | A | 5-10 | Private Research Centre | Unstable |
| 99 | 19 | MEDIUM | 11 | MEDIUM | 27 | A | 5-10 | Public Hospital | Unstable |
| 100 | 5 | LOW | 6 | MEDIUM | 20 | A | < 5 | Private Research Centre | Unstable |
| 101 | 11 | LOW | 0 | A | 16 | A | > 10 | Private Research Centre | Permanent |
| 102 | 25 | LOW | 1 | LOW | 31 | A | 5-10 | Public Hospital | Unstable |
| 103 | 26 | MEDIUM | 6 | MEDIUM | 41 | LOW | > 10 | Private Research Centre | Permanent |
| 104 | 8 | LOW | 3 | LOW | 36 | MEDIUM | < 5 | Public Hospital | Fixed-term |
| 105 | 4 | LOW | 1 | LOW | 29 | A | 5-10 | Public Hospital | Permanent |
| 106 | 23 | MEDIUM | 6 | MEDIUM | 33 | A | > 10 | Public Hospital | Permanent |
| 107 | 7 | LOW | 0 | LOW | 25 | A | < 5 | Public Hospital | Unstable |
| 108 | 14 | LOW | 1 | A | 22 | A | < 5 | Private Research Centre | Unstable |
| 109 | 23 | MEDIUM | 0 | LOW | 14 | A | > 10 | Public Research Centre | Unstable |
| 110 | 12 | LOW | 2 | LOW | 10 | A | < 5 | Private Research Centre | Unstable |
| 111 | 28 | MEDIUM | 10 | MEDIUM | 15 | MEDIUM | < 5 | Public Hospital | Permanent |
| 112 | 34 | A | 19 | A | 17 | A | 5-10 | Public Hospital | Unstable |
